# Orthostatic stress response in pediatric Fontan patients and the effect of ACE inhibition

**DOI:** 10.1371/journal.pone.0273940

**Published:** 2022-09-01

**Authors:** Lisette M. Harteveld, Nico A. Blom, J. Gert van Dijk, Robert H. Reijntjes, Paul J. van Someren, Fabian I. Kerkhof, Irene M. Kuipers, Lukas A. J. Rammeloo, Eco J. C. de Geus, Arend D. J. ten Harkel

**Affiliations:** 1 The Center for Congenital Heart Disease Amsterdam–Leiden, Leiden, The Netherlands; 2 Willem-Alexander Children’s Hospital, Department of Pediatrics, Division of Pediatric Cardiology, Leiden University Medical Center, Leiden, The Netherlands; 3 Department of Pediatric Cardiology, Amsterdam University Medical Centers, Amsterdam, The Netherlands; 4 Department of Neurology and Clinical Neurophysiology, Leiden University Medical Center, Leiden, The Netherlands; 5 Department of Biological Psychology, Faculty of Human Behavioral and Movement Sciences, Amsterdam Public Health Research Institute, Vrije Universiteit Amsterdam, Amsterdam, The Netherlands; Universita degli Studi Magna Graecia di Catanzaro, ITALY

## Abstract

**Background:**

Many cardiocirculatory mechanisms are involved in the adaptation to orthostatic stress. While these mechanisms may be impaired in Fontan patients. However, it is yet unclear how Fontan patients, who exhibit a critical fluid balance, respond to orthostatic stress. Angiotensin converting enzyme inhibitors are often prescribed to Fontan patients, but they may negatively influence orthostatic tolerance. Therefore, we evaluated the response to orthostatic stress in pediatric Fontan patients before and after treatment with enalapril.

**Methods:**

Thirty-five Fontan patients (aged 14 years) with moderate-good systolic ventricular function without pre-existent enalapril treatment were included. Before and after a three-month enalapril treatment period, the hemodynamic response to head-up tilt test was evaluated by various parameters including cardiac index, blood pressure, cerebral blood flow, aortic stiffness and cardiac autonomous nervous activity. Thirty-four healthy subjects (aged 13 years) served as controls.

**Results:**

Fontan patients had a decreased cerebral blood flow and increased aortic stiffness in the supine position compared to controls, while all other factors did not differ. Patients and controls showed a comparable response to head-up tilt test for most parameters. Twenty-seven patients completed the enalapril study with a mean dosage of 0.3±0.1mg/kg/day. Most parameters were unaffected by enalapril, only the percent decrease in cardiac index to tilt was higher after treatment, but the cardiac index during tilt was not lower (3.0L/min/m^2^ pre-enalapril versus 2.8L/min/m^2^ after treatment; *P* = 0.15).

**Conclusion:**

Pediatric Fontan patients adequately respond to orthostasis with maintenance of blood pressure and cerebral blood flow and sufficient autonomic response. Enalapril treatment did not alter the response.

**Clinical trial information:**

Scientific title: ACE inhibition in Fontan patients: its effect on body fluid regulation (sAFE-study).

The Netherlands National Trial Register: Trail NL6415. Registered 2017-07-20.

Trial information: https://www.trialregister.nl/trial/6415

## Introduction

The Fontan procedure is the current surgical palliative method for patients with a univentricular physiology. After Fontan surgery, both caval veins are directly connected to the pulmonary arteries, resulting in a circulation lacking a sub-pulmonary ventricle. Consequently, venous pressure must overcome pulmonary vascular resistance. Any increase in pulmonary vascular resistance will lead to increased central venous pressure resulting in venous congestion and decreased cardiac output [1]. As a result, the Fontan circulation has a delicate balance between systemic and pulmonary vascular resistance and a critical fluid balance [2]. Many internal or external factors may disrupt this balance, potentially resulting in circulatory impairment.

Investigating determinants of circulatory adaptation can be performed in a non-invasive, ambulatory setting by orthostatic stress testing as during orthostasis a cascade of cardiocirculatory mechanisms must be initiated to maintain an adequate circulation. This cascade starts by unloading of the baroreceptors in reaction to volume unloading, resulting in activation of the sympathetic nervous system, and concurrent vagal withdrawal, resulting in an increase in heart rate, systemic vasoconstriction, and venous return, to be able to maintain blood pressure (BP) and an adequate cerebral blood flow (3,4). In Fontan patients, many of these compensatory mechanisms have been described to be impaired, such as the cardiac autonomic nervous system activity (ANS), with decreased vagal- and increased sympathetic activity, and arterial vascular distensibility [3–7]. However, how Fontan patients respond to orthostatic stress, and if it will lead to circulatory impairment, remains incompletely understood. Thus far, the few small previous studies did not investigate cardiac autonomic and hemodynamic response simultaneously and studies that did investigate hemodynamic postural response have even shown differing results [8–11].

In addition, orthostatic stress testing can not only be used to unravel possible mechanisms of circulatory disorders, but can also be used to test the influence of various drugs on the circulatory system, such as angiotensin converting enzyme (ACE) inhibitors. ACE inhibitors are often prescribed in Fontan patients, despite the fact that their efficacy is controversial in these patients [12, 13]. In a biventricular circulation the use of ACE inhibitors may lead to orthostatic hypotension [14, 15] and in Fontan patients, with their critical fluid balance, these circulatory effects may be even more deleterious. However, the effect of ACE inhibitors on the orthostatic response in Fontan patients has not yet been investigated.

In the present study, we aimed to unravel the adaptation to orthostatic stress in pediatric Fontan patients by non-invasively examining the cardiovascular and autonomic response to head-up tilt testing (HUTT). In addition, we investigated the effect of enalapril treatment on the response to orthostatic stress in Fontan patients.

## Materials and methods

### Study population

Fontan patients between 8–18 years old, who were operated at the Leiden University Medical Centre were recruited from July 2017-October 2019. We included patients palliated with an extracardiac conduit and who had moderate-good systolic ventricular function to study a homogenous group. Patients with pre-existent ACE-inhibitors use and those who had difficulties to follow instructions were excluded from the study. Healthy children of similar age were recruited through advertisements at local schools to serve as controls. Written informed consent was obtained from all participants or their parents or guardians. The Medical Ethics Committee of the Leiden University Medical Center approved the study protocol.

### Study design

A combination of a cross-sectional and prospective intervention study was performed.

To test the reaction to orthostatic stress between the different groups, parameters were measured during supine rest and during HUTT. Therefore, in this study we investigated the cardiac ANS activity for the autonomic response, heart rate, BP and cardiac index for the hemodynamic response, peak hepatic vein flow and IVC collapsibility index for an estimation of change in systemic venous return and central venous pressure, and aortic stiffness parameters for the vascular response. Cerebral blood flow was measured as an end result. Furthermore, since respiratory parameters, including respiration rate, end-tidal CO2 (etCO2) and saturation, can influence or be affected by hemodynamic and ANS parameters during HUTT, we also evaluated the response of the respiratory parameters during this study.

At start of the study all participants were placed on a tilt table in supine position to first measure baseline parameters. After baseline measurements and at least 10 minutes of supine rest, a 60° HUTT on a mechanical tilt table with safety belts was performed. Tilt test could prematurely be terminated in case of imminent syncope or at patients request. All measurements performed in supine position were repeated after 3 minutes in the head-up tilt position as the acute phase has then passed and the parameters of interest have stabilized to the new hemodynamic situation.

After the first study day, Fontan patients started with three-months of oral enalapril treatment at a dosage of 5mg/day. Dosage was further titrated over a period of 2–3 weeks, according to BP measurements, to the target dose of 0.5mg/kg/day or a maximum of 20mg/day. If patients experienced side effects or if the systolic BP fell more than 20%, the dosage was lowered. After three months of treatment the HUTT with all measurements was repeated.

Echocardiographic assessment (for cardiac index and systemic venous parameters), BP and arterial stiffness measurements were performed once in each position. ANS activity, cerebral blood flow, etCO_2_, saturation and respiration rate were continuously monitored throughout the complete test; final values were obtained by averaging a period of 4 minutes in both positions. In the head-up tilt position the period 3–7 minutes after the start of tilt was taken.

### Echocardiography

Transthoracic echocardiography was performed on a Vivid S6/S60 (GE healthcare, Norway). Images were analyzed offline using EchoPac (version 203, GE healthcare). Pulse wave Doppler recordings across the (neo)aortic valve were performed to assess aortic velocity time integral from which, by using heart rate and (neo)aortic annulus diameter, cardiac index was calculated as follows: $\text{cardiac}\;\text{index}=\frac{\text{heart}\;\text{rate}*((\pi *{\left(\frac{\text{aortic annulus}}{2}\right)}^{2})*\text{velocity}\;\text{time}\;\text{intergal}))}{\text{body}\;\text{surface}\;\text{area}}$. In addition, Doppler recordings of the hepatic vein were performed to assess peak antegrade flow. Furthermore, the maximum and minimum diameter of the inferior vena cava (IVC) was measured by M-mode during a sniff-test from which we calculated the proportional change, the IVC collapsibility index. Averages of three measurements of each variable were used for analysis, if applicable.

### Arterial stiffness and blood pressure

Arterial stiffness, assessed by aortic pulse wave velocity (PWVao) and augmentation index (AIXao), and BP were measured using an oscillometric arteriograph device with the cuff on the left arm (Tensiomed, Hungary) [16]. The arteriograph software determines measurement accuracy with a standard deviation of the PWVao, based on at least two consecutive pulse waves. However, as children have smaller pulse waves and tilt leads to even smaller pulse waves, we checked each pulse wave to decide whether the result of the subsequent analysis could be accepted. If not, we analyzed the correct pulse waves separately and used the average of at least two pulse waves for analysis when the standard deviation of the PWVao <1.0m/s.

We initially recorded finger plethysmography (Finometer) data, but preliminary analysis revealed that the Modelflow method was not suitable to analyze the altered circulation in Fontan patients. These data will not be discussed further.

### Arterial cerebral blood flow

Mean velocity of both cerebri media arteries was assessed by transcranial Doppler sonography using a non-imaging doppler-Box with DWL Doppler system and QL software version 3.3 (Compumedics Germany, supplied by VCM medical, Netherlands) and two 2MHz probes attached to an adjusted Marc 500 headframe (Spencer Technology, America). For analysis, the artery with the highest mean velocity was used.

### Capnography and pulse oximetry

To measure blood oxygen saturation and etCO_2_, we used a Nihon Kohden (Japan) finger-clip pulse oximeter and a capONE mainstream CO_2_ sensor with a nasal/oral adapter.

### Cardiac autonomous nerve system activity and respiration

To measure cardiac ANS activity by heart rate variability parameters and measure respiration, impedance- and electrocardiogram registration was done using the VU-ambulatory monitoring system (VU-AMS; VU university, Netherlands, 5fs version). The VU-Data Analysis and Management Software (VU-DAMS, VU University) were used as described in previously published methods [17]. Before analysis, artefacts and ectopic beats were removed. Pre-ejection period (PEP), partly reflecting sympathetic inotropic effect [18], and respiratory sinus arrhythmia (RSA), mainly reflecting parasympathetic chronotropic effects [19], were calculated as described in previously published methods [17]. We additionally calculated the standard deviation of the inter-beat interval of normal sinus beats (SDNN) and the root mean square of successive differences between normal sinus beats (RMSSD), both reflecting parasympathetic activity on short-term recordings [20]. Furthermore, using the Fast Fourier Transformation we assessed low-frequency (LF;0.04–0.15Hz) and high-frequency (HF;0.15–0.4Hz) power and additionally calculated the LF:HF ratio, a disputed but frequently used measure to reflect sympathicovagal balance [21].

Both HR and respiration may affect cardiac ANS activity. It remains a subject of debate whether it is necessary to correct for these parameters. Some state HR adjustment might remove possible important variance of outcomes related to autonomic control [19]. Therefore, we have chosen to report HR and respiration in parallel with the heart rate variability parameters over adjustment procedures [19].

### Statistical analysis

Data analysis was performed using SPSS statistics (IBM, version 25). A *P*-value of 0.05 or less was considered as statistically significant. In order to perform reliable inference in the small study group, non-parametric tests were used for all comparisons. Categorical data are reported as numbers with percentages. Continuous data are presented as median with first to third quartile [Q1-Q3]. Comparison of baseline measurements during supine rest and percentage change of parameters during HUTT between Fontan patients and controls were performed with the Mann-Whitney *U* test. Furthermore, we compared the parameters of supine rest and HUTT within each group of subjects, Fontan patients and healthy controls, with the Wilcoxon Signed-Rank Test. The effects of enalapril treatment on baseline supine rest parameters and percentage change during HUTT were evaluated by using the Wilcoxon Signed-Rank Test as well.

## Results

**Fig 1:**
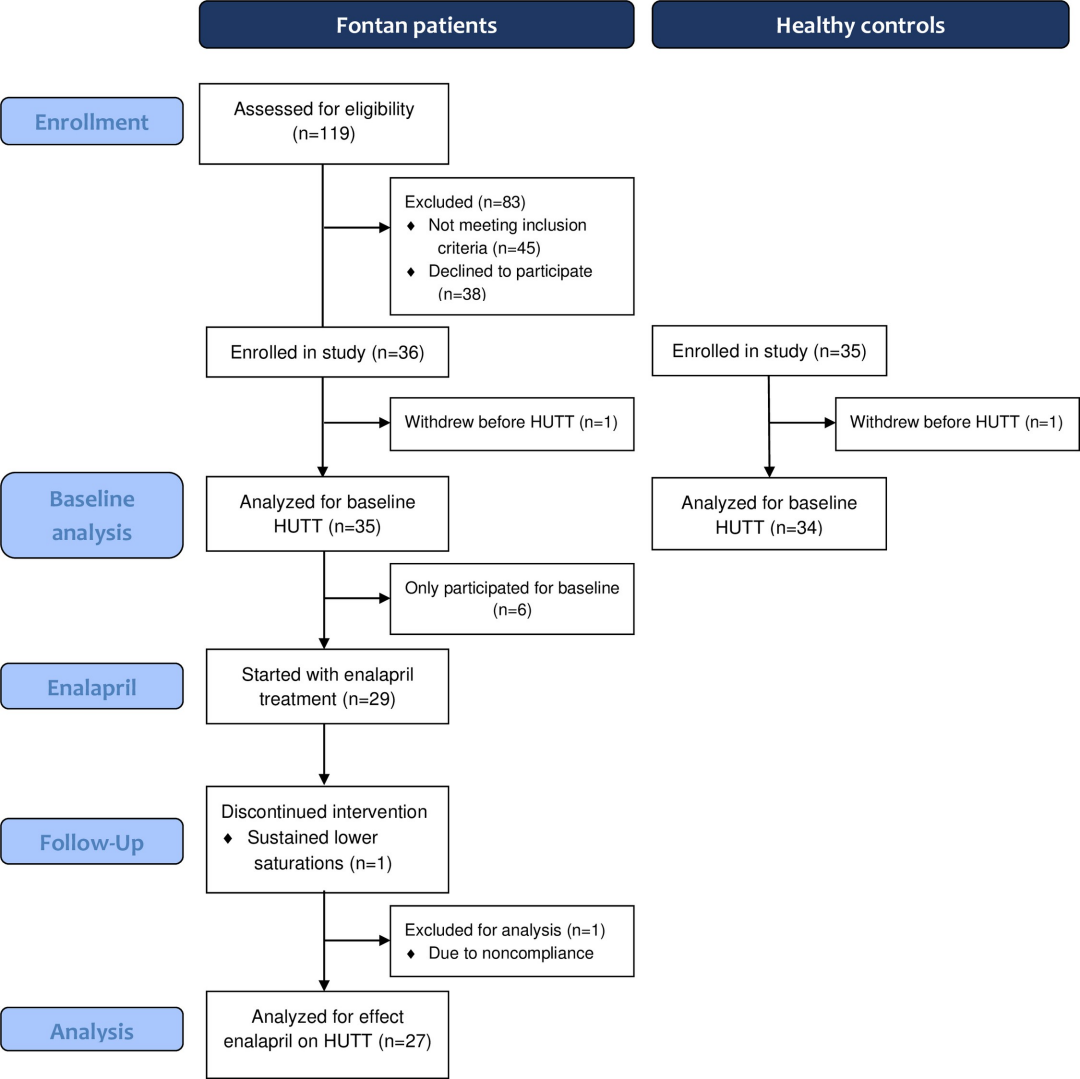
Flow diagram of the study. HUTT = Head-up tilt testing.

From 74 eligible Fontan patients for the study, 36 agreed to participate (49%). Thirty-five healthy controls were recruited. As one patient and one control withdrew before HUTT, 35 Fontan patients (median age 14.0 years) and 34 controls (median age 12.8 years) were analyzed (Fig 1). Patients who participated did not differ from those who did not in terms of age (median of 14.0 years [12.6–16.7] for participants vs. 13.0 years [11.7–16.0] for non-participants; *P* = 0.420) and morphology of the main single-ventricle (P = 0.303). Fontan patients did not differ from healthy controls in terms of baseline characteristics (Table 1). Twenty-one Fontan patients had a left (60%), 11 a right (31%) and 3 an indifferent dominant ventricle (9%). Mean age at Fontan operation was 3.2 years. A patent fenestration was present in one patient and one other had a pacemaker (dual chamber pacing; DDD). Fontan patients had a low plasma N-terminal pro brain natriuretic peptide (median 79ng/L). Although all Fontan patients had a subjective moderate to good systolic ventricular function and a comparable global longitudinal strain compared to healthy controls on echocardiography, Tissue Doppler imaging showed lower systolic velocities in Fontan patients compared to controls.

**Table 1:** Patient characteristics.

Characteristics	Fontan patients (N = 35)	Controls (N = 34)	*P*-value
Age (years)	14.0 [12.6–16.7]	12.8 [11.0–15.7]	0.193
Males (N,%)	23 (65.7)	17 (50.0)	0.186
Height (cm)	163.9 [153.6–173.0]	160.9 [148.9–170.0]	0.421
Weight (kg)	51.2 [42.5–60.0]	45.4 [36.4–56.2]	0.126
BSA (m^2^)	1.5 [1.38–1.72]	1.45 (0.3)	0.121
Diagnosis (N,%)			
Tricuspid atresia	9 (25.7)		
Pulmonary atresia	2 (5.7)		
Double inlet left ventricle	5 (14.3)		
Double outlet right ventricle	0 (0.0)		
Hypoplastic left heart syndrome	8 (22.9)		
Unbalanced atrioventricular septum defect	4 (11.4)		
Other	7 (20.0)		
Main ventricle (N;%)			
Left	21 (60.0)		
Right	11 (31.4)		
Indifferent	3 (8.6)		
Age at Glenn operation (years)	0.50 [0.38–0.76]		
Age at Fontan operation (years)	3.1 [2.8–3.6]		
Patent Fenestration (N,%)	1 (2.9)		
Pacemaker (N,%)	1 (2.9)		
NT-pro BNP (ng/L)	79.3 [45.5–136.1]		
Systolic ventricular function			
Global longitudinal strain (%)	15.2 [12.6–17.5]	16.3 [14.4–18.1]	0.056
TDI septal S’ (m/s)	0.043 [0.03–0.05]	0.080 [0.07–0.08]	<0.001
TDI lateral free wall systemic ventricle S’ (m/s)	0.060 [0.05–0.07]	0.107 [0.09–0.12]	<0.001
AV-valve regurgitation (N,%)			
No	16 (45.7)		
Mild	13 (37.1)		
Moderate	6 (17.1)		
Severe	0 (0.0)		
(Neo)Aortic regurgitation (N,%)			
No	30 (85.7)		
Mild	2 (5.7)		
Moderate	3 (8.6)		
Severe	0 (0.0)		
Cardiac medications (N, %)			
Acetylsalicylic acid	33 (94.3)		
Coumarin derivative	2 (5.7)		
β-blocker	1 (2.9)		
Diuretics	1 (2.9)		

Data expressed as n (%), mean (±SD), and median [Q1-Q3].

BSA = body surface area; NT-pro BNP = N-terminal pro brain natriuretic peptide; S’ = peak systolic TDI velocity, TDI = Tissue Doppler imaging.

During HUTT none of the Fontan patients had imminent syncope while 3 healthy controls (9%) did develop complaints. One control was excluded for analysis as the HUTT had to be stopped at 5 minutes from start HUTT and the other two were not excluded as they developed complaints after all measurements were finished. Cardiac index was calculated for 21 patients, as the aortic annulus could not be assessed in 14 patients. Due to an inadequate echo window, cerebral blood flow could be determined in 19 patients and 27 controls. Furthermore, seven patients were excluded from analysis of cardiac ANS activity due to atrial ectopy, nodal rhythm or pacemaker activity.

**Fig 2:**
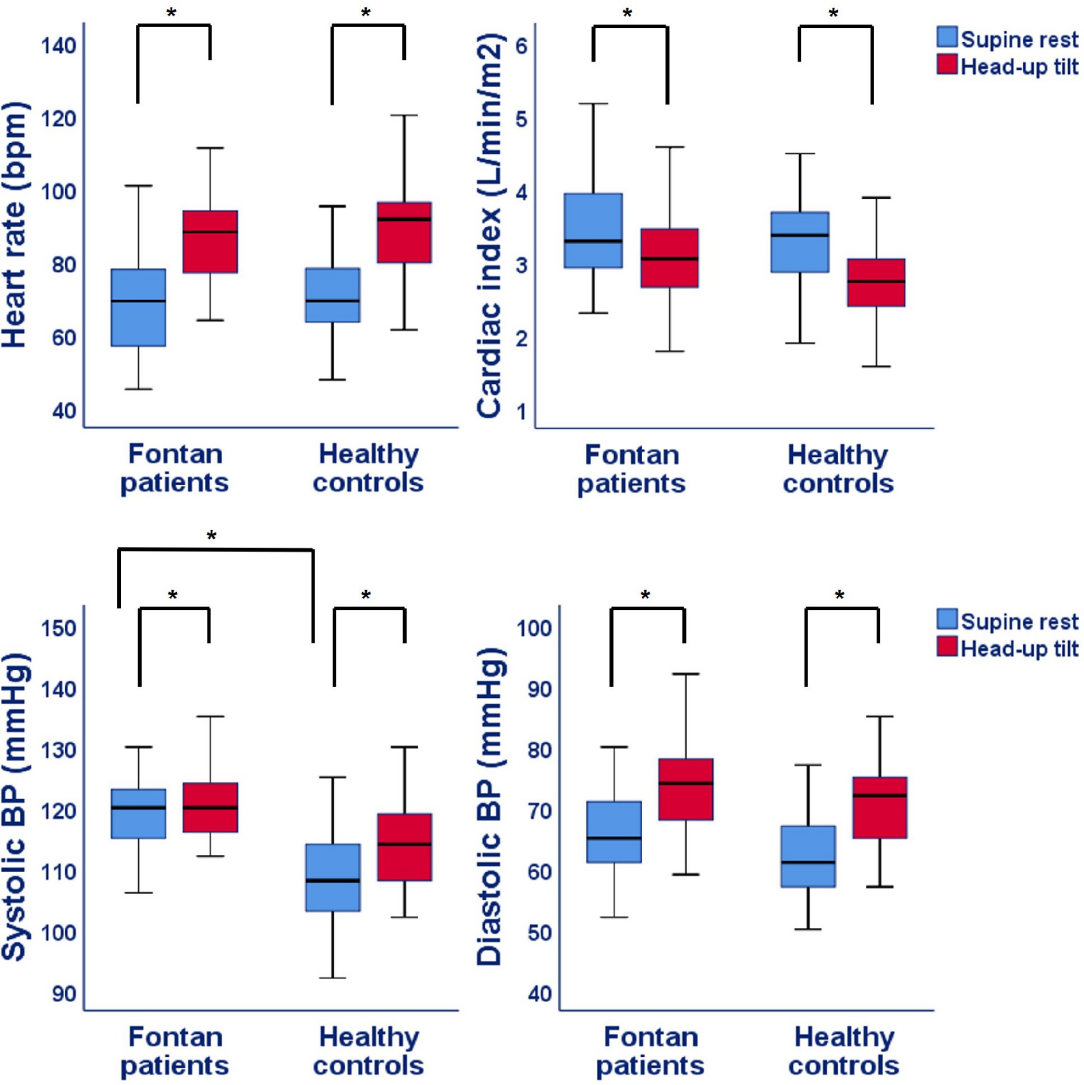
Hemodynamic parameters during supine rest and head-up tilt in Fontan patients and healthy controls. BP = Blood pressure; CI = Cardiac index; P-value: * <0.05 for difference in supine rest between Fontan patients and controls as well as difference between supine rest and head-up tilt within Fontan patients or healthy controls.

**Fig 3:**
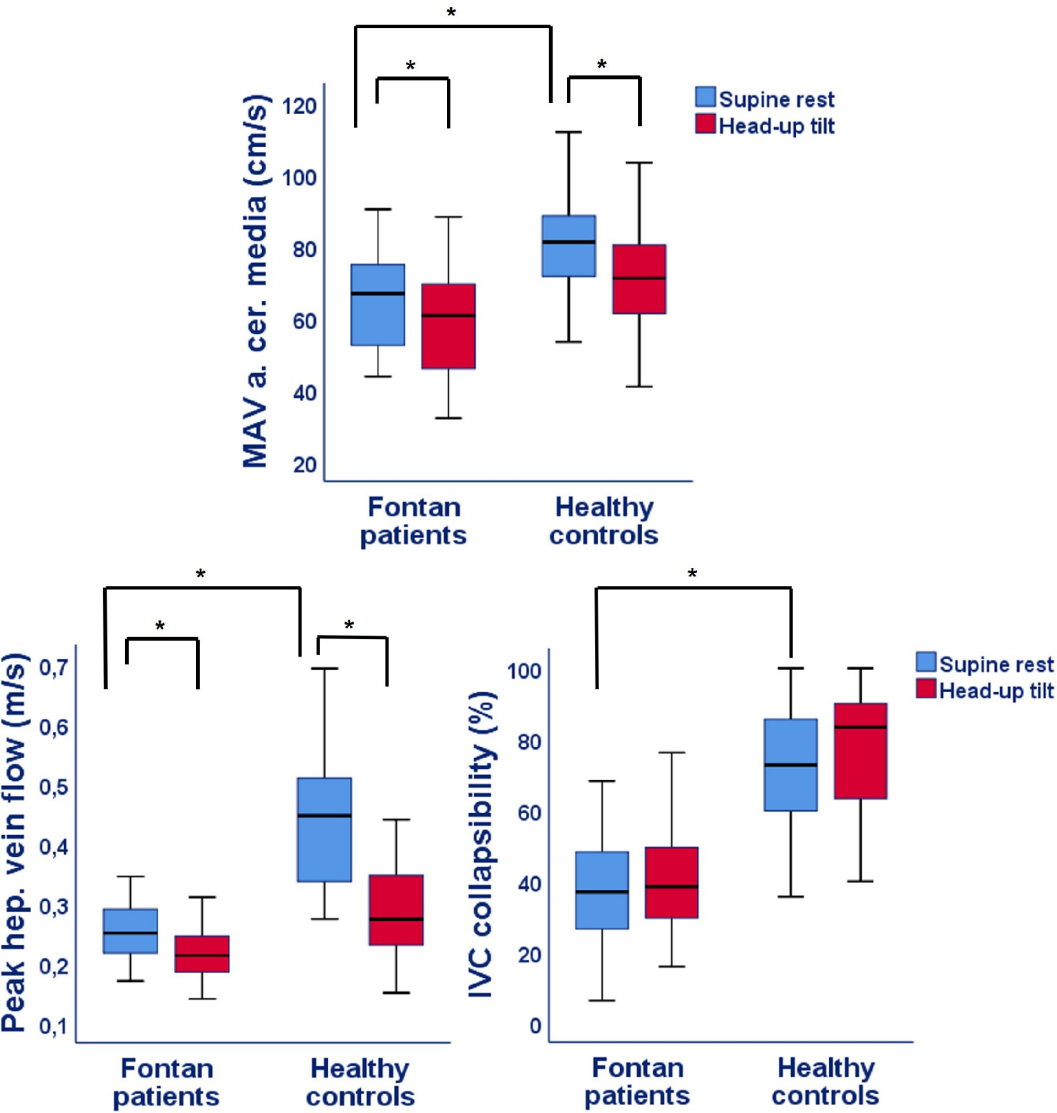
Mean arterial cerebral blood flow, peak hepatic vein flow and collapsibility index of the inferior vena cava in Fontan patients and healthy controls. IVC = Inferior vena cava; MAV = mean arterial velocity; P-value: * <0.05 for difference in supine rest between Fontan patients and controls as well as difference between supine rest and head-up tilt within Fontan patients or healthy controls.

**Fig 4:**
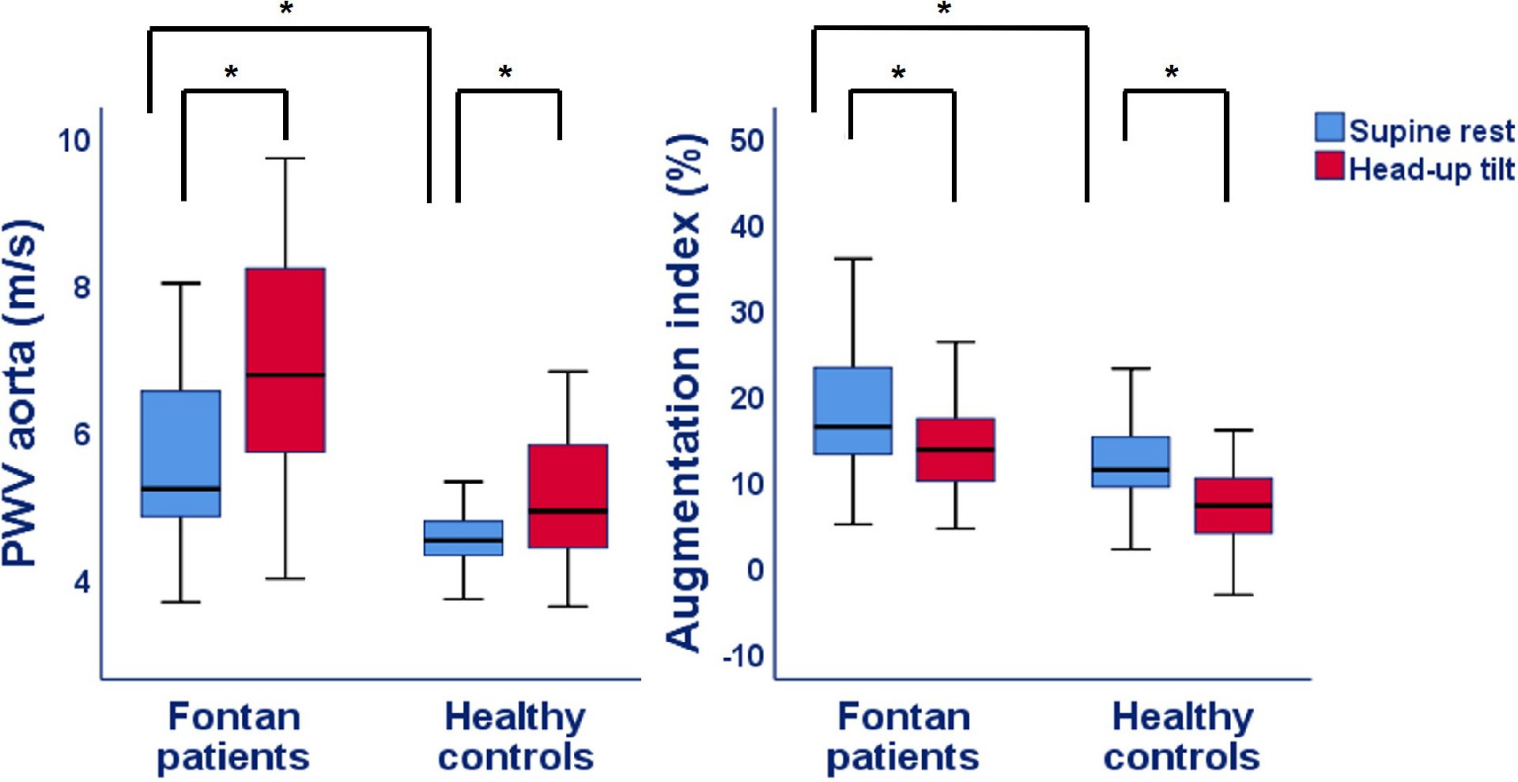
Aortic stiffness parameters during supine rest and head-up tilt in Fontan patients and healthy controls. PWV = Pulse wave velocity; P-value: * <0.05 for difference in supine rest between Fontan patients and controls as well as difference between supine rest and head-up tilt within Fontan patients or healthy controls.

**Fig 5:**
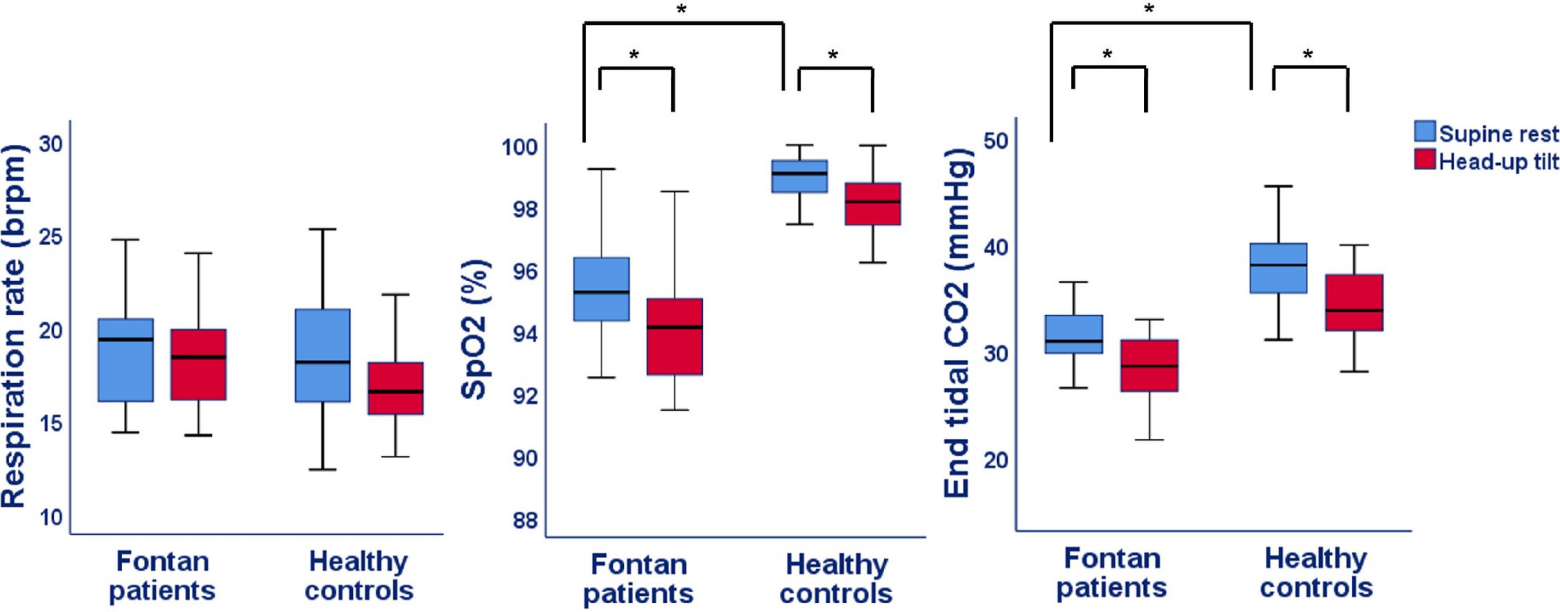
Respiration parameters during supine rest and head-up tilt in Fontan patients and healthy controls. brpm = breaths per minute; SpO2 = saturation; P-value: * <0.05 for difference in supine rest between Fontan patients and controls as well as difference between supine rest and head-up tilt within Fontan patients or healthy controls.

Figs 2–5 and Table 2 show the results of the supine and HUTT measurements in Fontan patients and healthy controls. At supine rest, patients had a similar cardiac index, but a lower cerebral blood flow and IVC collapsibility index compared to controls. Furthermore, both PWVao and augmentation index of the aorta were increased and etCO_2_ was decreased in the Fontan patients at rest. Although, parasympathetic activity was comparable between patients and controls at rest, PEP was significantly longer in Fontan patients, whereas the LF:HF ratio was higher.

**Table 2:** Cardiac autonomic nervous system activity parameters during supine and head up tilt in Fontan patients before enalapril treatment and at follow-up.

	Fontan patients		Healthy controls	
	Supine rest	Head-up tilt	Supine rest	Head-up tilt
RSA (ms)	70.0 [27.4–112.0]	35.3 [12.1–59.6] **ƗƗƗ**	84.5 [55.8–119.9]	47.7 [36.6–58.5] **ƗƗƗ**
RMSSD (ms)	60.6 [20.8–107.2]	25.3 [9.0–35.0] **ƗƗƗ**	62.5 [49.8–103.1]	30.0 [22.7–36.6] **ƗƗƗ**
SDNN (ms)	79.9 [28.0–126.1]	40.0 [19.2–54.5] **ƗƗƗ**	70.7 [55.1–97.6]	56.8 [44.8–70.6] **ƗƗ**
LF (ms^2^)	1259.5 [67–4555]	278.3 [67–826] **ƗƗ**	1229.2 [570–1788]	943.1 [585–1288]
HF (ms^2^)	944.1 [32–2467]	215.8 [19–462] **ƗƗƗ**	1234.9 [509–3045]	343.0 [214–546] **ƗƗƗ**
LF:HF	1.46 [0.8–2.4]	1.84 [1.3–3.3]	0.76 [0.4–1.4] *	2.62 [1.5–4.5] **ƗƗƗ**
PEP (ms)	124.2 [109.7–134.4]	133.3 [116.2–151.8] **ƗƗƗ**	82.2 [66.1–93.3]***	108.3 [103.0–119.3] **ƗƗƗ**

Data expressed as mean (±SD), and median [Q1-Q3].

P-value: * <0.05,

*** <0.001 for differences in supine rest between pre-enalapril and follow-up

P-value: ƗƗ <0.01, ƗƗƗ <0.001 for difference between supine rest and head-up tilt parameters in each group of subjects (pre-enalapril and follow-up)

HF = high frequency power spectral values; LF = low frequency power spectral values; LF:HF = low frequency/high frequency ratio; PEP = pre-ejection period; RMSSD = root mean square of successive differences between normal sinus beats; RSA = respiratory sinus arrhythmia; SDNN = standard deviation of the inter-beat interval of normal sinus beats.

During HUTT, most of the parameters increased or decreased with a similar magnitude in both groups (Table 3). However, the percentage increase of systolic BP (*P* = 0.019) and decrease of hepatic venous flow (*P* = 0.004) was lower in Fontan patients. The respiration rate decreased slightly more during HUTT in heathy controls (*P* = 0.013), while saturation decreased more in Fontan patients (*P* = 0.010). Furthermore, the increase of PEP during HUTT in Fontan patients was lower compared to controls (*P*<0.001), whereas the LF:HF ratio did not change in the patients (1.5 at rest vs 1.8 at tilt, *P* = 0.23), while in controls it increased (0.8 at rest versus 2.6 at tilt, *P*<0.001).

**Table 3:** Percentage change of parameters during head-up tilt in Fontan patients and healthy controls.

	Fontan patients	Healthy controls
*Hemodynamics*		
Heart rate (%)	+22.4 [13.9–44.0]	+30.0 [18.3–40.9]
Systolic BP (%)	+2.5 [-0.8–5.7]	+5.9 [+1.4–9.8] *
Diastolic BP (%)	+13.3 [1.5–21.8]	+14.5 [4.6–21.1]
Cardiac index (%)	-9.7 [-18.3–-4.6]	-18.0 [-24.6–-3.1]
*Cerebral blood flow*		
MAV art. cerebri media (%)	-6.4 [-14.0–-2.7]	-12.1 [-20.5–-4.0]
*Systemic venous parameters*		
Peak hepatic vein flow (%)	-14.6 [-28.9–0.0]	-31.3 [-47.0–-16.2] **
IVC collapsibility index (%)	+0.2 [-14.0–9.7]	+9.3 [-7.5–25.3]
*Arterial stiffness*		
PWVao (%)	+16.3 [13.7–38.6]	+16.1 [8.5–23.7]
AIXao (%)	-4.3 [-10.5–-1.2]	-6.3 [-9.1–-0.9]
*Respiration*		
Respiration rate (%)	-1.7 [-5.0–3.7]	-7.1 [-11.2–-3.4] *
SpO_2_ (%)	-1.8 [-2.5–-0.9]	-0.8 [-1.4–-0.1] *
EtCO_2_ (%)	-8.5 [-11.3–-5.8]	-7.8 [-12.2–-4.2]
*Cardiac ANS activity*		
RSA (%)	-44.0 [-62.7–-25.7]	-47.0 [-54.3–-31.8]
RMSSD (%)	-55.5 [-69.4–-27.1]	-52.7 [-68.4–-44.3]
SDNN (%)	-44.5 [-61.1–22.0]	-18.4 [-35.4–-3.2] **
LF (%)	-44.2 [-82.2–-15.6]	-5.6 [-46.9–53.0] *
HF (%)	-70.1 [-85.1–-24.6]	-71.3 [-81.2–-54.2]
LF:HF (%)	+26.1 [-34.5–158.5]	+228.7 [108.0–433.4] **
PEP (%)	+6.9 [0.3–16.9]	+31.6 [16.0–61.1] ***

Data expressed as median [Q1-Q3].

P-value: * <0.05,

** <0.01,

*** <0.001 for difference in percentage change

AIXao = Augmentation index of the aorta; BP = Blood pressure; bpm = beats per minute; EtCO_2_ = End-tidal carbon dioxide; HF = high frequency power spectral values; IVC = Inferior vena cava; LF = low frequency power spectral values; LF:HF = low frequency/high frequency ratio; MAV art. cerebri media = Mean arterial velocity arteria cerebri media; PEP = pre-ejection period; PWVao = Pulse wave velocity of the aorta; RMSSD = root mean square of successive differences between normal sinus beats; RSA = respiratory sinus arrhythmia; SDNN = standard deviation of the inter-beat interval of normal sinus beats; SpO_2_ = oxygen saturation.

Twenty-nine Fontan patients started with enalapril treatment (Fig 1). One patient withdrew as a result of sustained lower saturations and one patient was excluded for further analysis due to non-compliance. Ten patients could not reach the targeted dosage due to a decrease in systolic blood pressure (n = 6, 22%) or other adverse events, consisting of syncope (n = 2), dizziness (n = 1) and palpitations (n = 1). Furthermore, one patient reported a hacking cough after completion of the study which disappeared after discontinuation of enalapril. The remaining 27 patients completed the treatment period with a mean dosage of 0.3±0.1mg/kg/day.

The effects of enalapril treatment on all cardiovascular parameters studied in this study during supine rest are depicted in Table 4. Enalapril treatment lowered mean systolic (*P* = 0.006) and median diastolic BP (*P* = 0.03) at supine rest, however, no other parameters at rest were affected by enalapril. Furthermore, after enalapril treatment AV-valve regurgitation changed in only one patient and improved from moderate to mild.

**Table 4:** Comparison of baseline measurements during supine rest between Fontan patients before enalapril treatment and at follow-up.

	Pre-enalapril	Follow-up
*Hemodynamics*		
Heart rate (bpm)	63.1 [54.1–76.6]	63.3 [53.7–82.1]
Systolic BP (mmHg)	120.0 [111.5–124.0]	112.0 [109.0–121.5] **
Diastolic BP (mmHg)	65.0 [60.5–70.5]	61.0 [59.0–65.5] *
Cardiac index (L/min/m^2^)	3.2 [2.9–3.5]	3.3 [2.9–4.1]
*Cerebral blood flow*		
MAV art. cerebri media (cm/s)	59.7 [50.8–69.0]	56.9 [48.8–67.6]
*Systemic venous parameters*		
Peak hepatic vein flow (m/s)	0.26 [0.21–0.30]	0.28 [0.23–0.34]
IVC collapsibility index (%)	38.4 [29.6–56.2]	38.1 [26.4–46.5]
*Arterial stiffness*		
PWVao (m/s)	5.2 [4.9–6.2]	5.3 [4.3–5.8]
AIXao (%)	17.2 [13.4–23.9]	18.7 [13.8–21.6]
*Respiration*		
Respiration rate (brpm)	19.4 [16.7–21.5]	19.3 [16.6–19.9]
SpO_2_ (%)	95.2 [94.4–96.4]	95.1 [94.2–96.0]
EtCO_2_ (mmHg)	30.6 [29.5–32.9]	30.4 [28.2–32.3]
*Cardiac ANS activity*		
RSA (ms)	81.5 [42.2–111.1]	66.7 [35.7–88.2]
RMSSD (ms)	78.0 [40.8–110.6]	68.8 [30.0–94.8]
SDNN (ms)	86.9 [46.4–132.4]	79.2 [43.0–100.7]
LF (ms^2^)	1464.0 [339–4648]	1495.3 [336–2835]
HF (ms^2^)	1062.5 [209–2690]	1084.0 [221–2150]
LF:HF	1.6 [0.8–2.5]	1.4 [0.9–2.3]
PEP (ms)	124.9 [110.9–137.8]	131.3 [113.5–139.4]

Data expressed as median [Q1-Q3].

P-value: * <0.05,

** <0.01 for difference in percentage change

For previously used abbreviations see Table 3; brpm = breaths per minute

Table 5 shows the results of percentage change during HUTT on Fontan patients before enalapril treatment and after enalapril treatment. Although most changes induced by HUTT were similar before as well as after enalapril treatment, cardiac index decreased significantly more during tilt with enalapril treatment (*P* = 0.03). However, the mean cardiac index during tilt after treatment was not significantly lower than during tilt before treatment (3.0/min/m^2^ before enalapril treatment versus 3.0 L/min/m^2^ at follow-up; *P* = 0.13).

**Table 5:** Percentage change of parameters during head-up tilt in Fontan patients before enalapril treatment and at follow-up.

	Pre-enalapril	Follow-up
*Hemodynamics*		
Heart rate (%)	+25.7 [18.9–44.2]	+27.0 [17.9–38.9]
Systolic BP (%)	+3.2 [0.0–7.3]	+3.3 [-3.1–10.5]
Diastolic BP (%)	+17.2 [4.0–24.0]	+11.1 [8.9–21.5]
Cardiac index (%)	-6.7 [18.2–2.4]	-20.5 [-27.6–-6.8] *
*Cerebral flow*		
MAV art. cerebri media (%)	-5.1 [21.8–1.2]	-2.4 [-18.0–3.8]
*Systemic venous parameters*		
Peak hepatic vein flow (%)	-21.0 [-29.3–-3.6]	-13.6 [-34.9–1.1]
IVC collapsibility index (%)	+0.2 [-14.6–24.2]	+5.8 [-6.2–24.1]
*Arterial stiffness*		
PWVao (%)	+21.2 [13.8–38.9]	+24.4 [15.5–36.8]
AIXao (%)	-4.3 [-10.5–-1.2]	-2.9 [-10.0–-0.5]
*Respiration*		
Respiration rate (%)	-3.0 [-6.4–2.0]	-3.2 [-12.0–3.4]
SpO_2_ (%)	-2.0 [-2.5–-0.9]	-1.5 [-2.9–-0.7]
EtCO_2_ (%)	-7.3 [-10.8–-5.6]	-10.1 [-13.4–-5.8]
*Cardiac ANS activity*		
RSA (%)	-44.0 [-65.5–-12.3]	-34.3 [-61.9–-8.6]
RMSSD (%)	-57.6 [-76.0–-28.8]	-52.2 [-71.1–-27.4]
SDNN (%)	-47.6 [-64.5–-25.5]	-27.1 [-61.6–-5.8]
LF (%)	-49.5 [-89.6–-18.0]	-62.7 [-88.0–-25.0]
HF (%)	-70.2 [-89.6–-22.2]	-73.4 [-86.6–-51.6]
LF:HF (%)	+13.6 [-37.5–130.7]	+60.9 [-18.9–181.7]
PEP (%)	+6.9 [-0.2–19.5]	+5.6 [-1.3–20.0]

Data expressed as median [Q1-Q3].

P-value: * <0.05 for difference in percentage change

For used abbreviations see Table 3.

## Discussion

Our study demonstrated that Fontan patients responded adequately to orthostatic stress, with normal response to HUTT for most parameters, including BP, cardiac index, aortic stiffness and vagal activity. Furthermore, enalapril treatment resulted in a lower BP, but did not hamper the response to orthostatic stress.

HUTT is generally used to test orthostatic tolerance of syncope patients [22]. During HUTT, redistribution of blood volume to the lower body occurs which results in unloading of the baroreceptors [23]. This activates the sympathetic nervous system and causes concurrent vagal withdrawal, to increase heart rate, systemic vasoconstriction and venous return and maintain BP and cerebral blood flow [23, 24]. When these compensating mechanisms are impaired, arterial pressure will decrease, resulting in a decrease of BP and, when cerebral circulatory mechanism also fail, insufficient cerebral blood flow to maintain consciousness [24].

The cardiocirculatory resting condition from Fontan patients differed from that of healthy controls, so a different response to HUTT might be expected. At supine rest, patients had a lower IVC collapsibility index, which most likely is the result of venous pooling, necessary to increase central venous pressure to an extent that it may function as the driving force to push blood through the pulmonary circulation. An increased arterial stiffness at rest has previously been described in Fontan patients and may reflect vascular dysfunction and increased sympathetic activity [10]. The lower cerebral blood flow in Fontan patients at rest probably is the result of increased aortic stiffness and central venous pressure combined with cerebral vasoconstriction induced by the lower etCO_2_ [25, 26]. The origin of decreased etCO_2_ is as yet unclear, although thoracic impedance is described to be reduced after thoracotomy, influencing pulmonary efficiency [27].

An adequate response to HUTT necessitates integrated responses by the autonomic nervous system, vascular function, regulation of cerebral blood flow. Previous studies have shown a reduced baroreflex sensitivity and markedly impaired cardiac ANS activity in Fontan patients, reflected in an increased sympathetic and decreased parasympathetic activity, however, the majority of these studies were performed in adult patients [3, 5, 10, 11]. In contrast to these studies, we showed that parasympathetic activity was not impaired during resting conditions and showed a normal response upon HUTT. The LF:HF ratio did not increase further upon HUTT but was already increased in supine position which could indicate that sympathetic activity was already at the upper end in resting condition [28]. However, the LF:HF ratio as sympathicovagal balance is controversial as LF reflects both para- and sympathetic activity. Furthermore, PEP, another parameter of sympathetic activity was increased as well in Fontan patients, suggesting a decreased sympathetic activity. However, PEP is also influenced by changes in pre- and afterload and is therefore a less reliable parameter of sympathetic activity in Fontan patients and also when used during postural changes. During HUTT, PEP increased in both groups while a decrease was expected due to the known increase in sympathetic activity. However, this response of PEP to postural change is consistent with previous studies and is due to a decrease in preload and an increase in afterload [29, 30]. The decreased preload leads to a longer PEP via a decrease of end-diastolic volume which results in a decrease of strength of cardiac contraction mediated by the Frank-Starling mechanism. The increased afterload prolongs the PEP, which ends at aortic valve opening, because it takes longer for the ventricular pressure to build up to the level of the aortic pressure.

Although sympathetic activity could not be reliably measured, our Fontan patients showed an adequate HR response to orthostasis with a similar increase in heart rate as in healthy controls indicating an intact cardiac autonomic regulation. An adequate vagal response to orthostatic stress in Fontan patients conflicts with one previous study investigating parasympathetic activity during standing in 8 adult patients showing a blunted parasympathetic response [11]. Age may have influenced this difference in results, as impaired autonomic nervous system activity has been demonstrated in mainly adult Fontan patients.

Aortic stiffness increased normally during tilt in our patients, despite it was already increased at rest, as illustrated by an increase in PWVao, a direct measure of stiffness unaffected by heart rate and stroke volume as is known from augmentation index [31]. The increase in stiffness during orthostatic stress confirms the result of two previous small studies, with respectively 8 and 18 adult Fontan patients, where an increase in systemic vascular resistance and plasma norepinephrine were found [8, 10].

Cerebral blood flow decreased in both groups in reaction to tilt, but sufficient flow was maintained in all Fontan patients since none of the patients developed syncope, even despite a lower cerebral blood flow in Fontan patients at rest. Cerebral blood flow during tilt is not only directly affected by a lowering of the cardiac index, but a decrease in cardiac index also causes a decrease in etCO_2_, resulting in cerebral vasoconstriction [32, 33].

IVC collapsibility index in Fontan patients remained low during tilt. Patients seem to adapt to the Fontan circulation to prevent venous pooling while standing and avoid a deleterious drop in central venous pressure and cardiac index. As observed by a study of Krishnan et al. [9], venous pooling might be prevented by an increase in venous tone and peripheral arterial resistance, especially in the legs, in Fontan patients which results in decreased venous capacitance and compliance. The integrity of the regulating mechanisms is further underscored by a similar change in cardiac index in Fontan patients as compared to controls. In older Fontan patients a larger decrease in cardiac output upon tilt has been demonstrated, suggesting that the adaptive mechanisms may be falling short during long-term follow-up [10].

A further challenge for adaptive mechanisms to tilting occur after enalapril treatment. Enalapril treatment resulted in a lower BP at rest but did not lead to orthostatic hypotension. Although orthostatic hypotension has been described as a side effect of ACE inhibitors, it usually occurs only as a first-dose phenomenon or during treatment in conjunction of dehydration or other hypotensive drugs [14, 15]. Furthermore, the percentage decrease of cardiac index was higher after treatment with enalapril, however the cardiac index during tilt was not significantly lower as compared with values before treatment. Previous studies performed in normal male subjects and hypertensive patients also showed that ACE inhibition did not result in a lower cardiac index during HUTT [34, 35]. The vasodilator effect of ACE inhibitors is apparently minimal, otherwise vasodilation would have resulted in increased venous pooling and respectively a decreased central venous pressure and cardiac index, which could be particularly detrimental in Fontan patients.

In clinical perspective, this study demonstrated that well-functioning asymptomatic patients at a relatively early stage after the completion of the complex Fontan surgery have a normal response to orthostatic stress comparable to healthy controls. However, as it is known that the Fontan circulation will deteriorate on the long term, ultimately resulting in Fontan failure, Fontan patients may respond abnormally at a later stage. The response to orthostatic stress testing may be useful to distinguish underlying mechanisms of Fontan failure during follow-up. In addition, it may help to evaluate the effects of heart failure medication in patients with an impaired Fontan circulation.

This study had some limitations. We included pediatric patients with an extracardiac conduit and moderate-good systolic ventricular function and therefore created a selection bias. However, our aim was to investigate a more homogenous group of asymptomatic patients to comprehend the adaptive mechanisms of a well-functioning Fontan circulation which may form a basis for further research to elucidate the development of Fontan failure. Our results are limited to the investigated population and could not be generalized. Furthermore, the non-invasive study set-up has its limitations as, for example, venous pressure could not be determined and might have completed the understanding of the adaptive mechanisms. Finally, sympathetic activity could not be directly determined from our measurements and more invasive measurements of sympathetic activity are necessary in future research.

## Conclusion

Pediatric patients with a Fontan circulation can respond adequately to orthostatic stress and maintain adequate BP, cardiac output and cerebral blood flow. We found no evidence of autonomic circulatory impairment. Enalapril treatment resulted in a lower BP at rest but did not alter the response to orthostatic stress and did not lead to orthostatic hypotension or a lower cardiac index during HUTT.

## Supporting information

S1 Checklist (PDF)

S1 Dataset (XLSX)

S1 Protocol (PDF)
