# Supplementary material for: Orthostatic stress response in pediatric Fontan patients and the effect of ACE inhibition
Source: PLoS One. 2022 Sep 1;17(9):e0273940. doi: 10.1371/journal.pone.0273940 (PMC9436155; doi:10.1371/journal.pone.0273940)
Supplement: S1 Protocol — (PDF) [file pone.0273940.s003.pdf]

# *ACE inhibition in Fontan patients: its effect on body fluid regulation*

# *(SAFE trial)*

**Principal investigators**
Dr. A.D.J. ten Harkel
Department of pediatric cardiology
Albinusdreef 2, 2333 ZA Leiden
Tel: +3171-5262835
E-mail: A.D.J.Ten_Harkel@lumc.nl

Prof. Dr. N.A. Blom
Department of pediatric cardiology
Albinusdreef 2, 2333 ZA Leiden
Tel: +3171-5262833
E-mail: N.A.Blom@lumc.nl

Dr A.A.W. Roest
Department of pediatric cardiology
Albinusdreef 2, 2333 ZA Leiden
Tel: +3171-5262835
E-mail: A.Roest@lumc.nl

Prof Dr. J.C.N. de Geus
Department of Biological Psychology / EMGO+ institute, VU University
van der Boechorststraat 1, 1081 BT Amsterdam
Tel: +3120-5988813
E-mail: eco.de.geus@vu.nl

Prof. Dr. J.G. van Dijk
Department of neurology
Albinusdreef 2, 2333 ZA Leiden
Tel: +3171-5263960
E-mail: J.G.van_dijk@lumc.nl

**PhD Student**
L. M. Harteveld
Department of pediatric cardiology
E-mail: L.M.Harteveld@lumc.nl

**Independent expert**
Drs. E.G.J. Rijntjes-Jacobs
Department of neonatology
Tel: +3171-5264132
E-mail: E.G.J.Rijntjes-Jacobs@lumc.nl

**Pharmacy**
Clinical Pharmacy and Toxicology Leiden University Medical Centre (Hospital Pharmacy LUMC)
Tel: +3171-5262790
E-mail: Trials@lumc.nl

# Table of Contents

1. Introduction and Rationale ..... 4
2. Objectives ..... 8
3. Study design ..... 9
4. Study population ..... 10
Population: ..... 10
Inclusion criteria: ..... 10
Exclusion criteria: ..... 10
Sample size calculation: ..... 10
5. Treatment of subject: ..... 11
6. Study parameters ..... 12
Secondary study parameters: ..... 12
7. Data analysis ..... 14
Statistics ..... 14
8. Expected results ..... 15
9. Administrative aspects of participants data ..... 16
Confidentially of participants data ..... 16
Access to data of participants ..... 16
Retention period of participants data ..... 16
Information about unexpected results ..... 16
Withdrawal of consent ..... 16
More information about rights and processing of participants data ..... 17
Registration of this research ..... 17
10. References ..... 18

**SUMMARY**

**Rationale:** There is no consensus on the use of ACE inhibition in Fontan patients without ventricular dysfunction. Multiple centres prescribe enalapril on a routine base for patients with a Fontan circulation and a preserved ventricular function, while other centres have considerable doubt about its effectiveness. Too little research has been done to the effectiveness of ACE inhibition in Fontan patients. There are as yet no studies available that investigate the effect of ACE inhibition on various cardiovascular parameters in patients with a Fontan circulation. By studying the effect of ACE inhibition on cardiovascular parameters as systolic and diastolic ventricular function, cardiac output, and the sensitivity of the cardiovascular system to fluid changes, the basic effects of ACE inhibition on the cardiovascular system of Fontan patients will become more clear. This will result in a more appropriate selection of patients that will profit of the use of ACE inhibitors.

**Main objective**: To treat Fontan patients for 3 months with the ACE inhibitor enalapril and compare a set of cardiovascular measurements before and after treatment in order to study its effect on the cardiovascular system and the effect of a reversible fluid challenge and depletion in Fontan patients, and to correlate all these results with the results of a symptom limited maximal exercise test.

**Study design:** This study consists of a longitudinal intervention study and a cross-sectional study.

**Study population:** 55 patients with a univentricular heart after palliation with the Fontan circulation will be included from an age of 8 until 18 years old. Patients who already use enalapril will be excluded. A number of fifty healthy age and gender matched subjects will serve as controls.

**Intervention (if applicable)**: To all Fontan patients enalapril will be given twice daily at a dose of 0,5 mg/kg/day with a maximum of 20 mg per day. All Fontan patients will undergo all the investigations before and after treatment and healthy controls will undergo all the investigations once, except for blood testing and the symptom limited exercise test which will not be performed in healthy controls.

**Main study parameters/endpoints:**
- Cardiopulmonary exercise stress test: $VO_2$peak.
- Cardiac autonomic nervous activity: heart rate variability and pre-ejection period.
- Outcome of passive leg raising and head up tilt table testing: cardiac output and cardiac autonomic tone.

**Nature and extent of the burden and risks associated with participation, benefit and group relatedness:** Patients with a Fontan circulation will have to visit the LUMC twice and these visits will be instead of normal appointments. In the future, Fontan patients could possibly have advantage from this research. For healthy subjects, this research means that they have to come only once to the LUMC. The exams themselves are not harmful to health, will cost three hours per visit and have a low burden. Only venapunction, which only will be done in the group of Fontan patients, can be painful, but we will thrive to soften the pain by using, for example, emla cream.

**1. Introduction and Rationale**

At present more than 90% of patients with congenital heart disease reach adulthood. However, their life expectancy is less than normal, and many patients suffer from heart failure or pulmonary hypertension (Nieminen et al). A group of patients that is especially prone to these long-term sequelae are patients with a univentricular heart. Although heart failure may be treated medically by anti-heart failure medication, most studies evaluating the effectiveness of these medications are performed in patients with a biventricular circulation.

Patients with a univentricular heart usually undergo a stepwise Fontan procedure. This technique consists of a stepwise approach with first connecting the superior vena cava with the pulmonary arteries and at a later stage connecting the inferior vena cava with the pulmonary arteries, either as an intra-atrial tunnel, or as an extra cardiac conduit (Total Cavopulmonary Connection; TCPC). After Fontan palliation these patients lack a heart chamber that pumps the blood through the pulmonary circulation. Therefore venous pressure is necessary to overcome pulmonary resistance. Any increase in pulmonary vascular resistance will lead to increased central venous pressure and venous congestion. On the other end of the circulation there is downstream decreased flow (Gewillig et al). This results in a critical fluid balance and dependence of cardiac output on adequate preload (La Gerche et al). While a volume challenge can increase cardiac output in patients (De Mey et al), fluid overload can lead to increased central venous pressure, resulting in venous congestion and ascites and pleural effusion, while dehydration immediately has a negative effect on cardiac output and may result in signs of clinical forward failure. In Fontan patients there is a delicate balance between systemic and pulmonary vascular resistance, while systemic circulation is highly preload dependent. In most Fontan patients a fluid challenge resulted in an increase in end-diastolic pressure and an increase in cardiac output as well (De Mey et al). On the other hand, an increase in end-diastolic pressure may have detrimental effects on the Fontan circulation due to a decrease of the transpulmonary gradient.

Fontan patients show a slightly diminished diastolic and systolic ventricular function already 10 years after completion of the TCPC (Bossers et al). During adulthood an increasing number of patients will suffer from clinical signs of ventricular dysfunction (Ohuchi J Cardiol 2016). Furthermore, a decrease in aortic distensibility is now being recognized as an important factor for increased afterload of these ventricles (Ohuchi et al), thereby increasing the process of ventricular deterioration.

Furthermore, the normal mechanisms that can counteract the negative effects of changes in fluid volume have been described to be diminished in Fontan patients as well. The main regulatory mechanisms in healthy individuals to counteract the effects of fluid changes on cardiovascular

parameters are the possibility of the heart to change stroke volume, the autonomic nervous system and the aortic stiffness or aortic distensibility.

Ventricular function:

During longer-term follow-up a significant number of patients show impaired ventricular function (Ono et al). When dobutamine stress is used in Fontan patients even those with a normal ventricular function lack the possibility to increase their stroke volume (Robbers-Visser et al). These cardiovascular changes in most patients result in a diminished exercise capacity (Bossers et al 2014).

Cardiac autonomic Nervous Activity:

Failure of the autonomic nervous system to adapt to differing circumstances is one of the key markers in the development of heart failure and may result in an increased risk of rhythm disturbances (Dekker et al 1997; La Rovere et al 2001; Nolan et al 1998; Saul et al 1988; Schwartz et al 1992). Assessment of cardiac autonomic function has important clinical implications as cardiac autonomic balance can be positively modified by exercise training (Groehs et al 2015) and a variety of other pharmacological and more invasive measures (Schwartz et al 2015), contributing to improvement of prognosis. Reports in patients with congenital heart disease including Fontan patients have shown a reduction in cardiac autonomic nervous activity, although its relation to symptoms and prognosis remains as yet unclear (Ohuchi et al 2003; Ohuchi et al 2011). In Fontan patients parasympathetic activity seems to be reduced as assessed by the heart rate recovery after cessation of exercise. In addition, various aspects of heart rate variability (HRV) are reduced in Fontan patients, reflecting both parasympathetic as well as sympathetic cardiac autonomic nervous activity (Bossers et al 2015). The clinical value of HRV in Fontan patients has yet to be established, but it has been demonstrated previously that HRV analysis might contribute to early detection of patients who will develop arrhythmias (Dahlqvist et al 2011).

Aortic stiffness:

In addition to reduced ventricular function, in many congenital heart disease patients, the vascular function of especially the aortic arch show significant differences from normal (Kojima et al). Several studies have investigated vascular function in Fontan patients. Both in paediatric patients as well as in adult patients arterial stiffness and pulse wave velocity are increased (Tomkiewicz et al; Lambert et al; Biglione et al; Myers et al). However, no clear relation with ventricular function could be made. In the assessment of cardiovascular risk factors the measurement of pulse wave velocity of the aorta has become a key factor. Aortic pulse wave velocity is a surrogate of aortic stiffness.. Since arterial baroreceptors are present in the aortic arch it is understandable from a physiological standpoint that arterial stiffness and cardiac autonomic function have a relationship. Various studies have found a

correlation between cardiovascular autonomic function and indices of arterial stiffness (Chrysohoou et al; Nemes et al).

In the present study we will evaluate 46 patients with functional univentricular hearts after TCPC. A fluid challenge will be given by passive leg raising. Passive leg raising has shown to be an effective and reversible and safe method of studying the effect of a fluid challenge on various cardiovascular parameters (Cherpanath et al). This method is extensively being used in intensive care units to predict the effect of fluid challenge on the circulation of various groups of patients. Depletion of central and splanchnic blood volume will be induced by a passive head-up-tilt test. In a previous study the reduction of portal venous flow is more pronounced in symptomatic Fontan patients (Hsia et al. J Thorac Cardiovasc Surg).

Enalapril:

Enalapril, an angiotensin converting enzyme (ACE) inhibitor, has been shown to be effective in reducing mortality in patients with heart failure and reduced systolic function for more than 25 years. In subsequent studies its effectiveness has also been proven in patients with mild to moderate symptomatic heart failure. In recent guidelines ACE inhibition has therefore become the cornerstone of therapeutic interventions in patients with systolic heart failure. The effectiveness of ACE inhibition has been ascribed to its effect on various cardiovascular and pulmonary parameters, including systemic vascular resistance, cardiac autonomic tone (KD Maida et al), aortic pulse wave velocity (PWV) and lung function (Contini et al; Abraham et al). The success of ACE inhibition in patients with systolic heart failure and a biventricular heart has also led to the introduction of ACE inhibition to improve the Fontan circulation in patients with a univentricular heart.

The use of ACE inhibition in patients with a univentricular heart is, however, controversial. There is consensus on the use of ACE inhibition in Fontan patients with ventricular dysfunction. However, many centres also prescribe enalapril on a routine base for patients with a Fontan circulation and a preserved ventricular function, while other centers have considerable doubt about its effectiveness (Wilson et al; Int J Cardol; Wilson et al Heart Lung and circulation). In a small study no effect of enalapril on exercise capacity in Fontan patients has been found (Kouatli et al, circulation). However, when enalapril is used in univentricular pre-Fontan patients a decrease in end-diastolic pressure (Yim et al Cardiol Young) and a redirection of blood flow to the systemic circulation (Lee et al; Heart 2011) has been found. These cardiovascular effects, however, did not improve somatic growth, ventricular function or heart failure severity score during the first year of life (Hsu et al Circulation 2010).

There are as yet no studies available that investigate the effect of ACE inhibition on various cardiovascular parameters in patients with a Fontan circulation. By studying the effect of ACE inhibition on cardiovascular parameters as systolic and diastolic ventricular function, cardiac output, and the sensitivity of the cardiovascular system to fluid changes the basic effects of ACE inhibition on the cardiovascular system of Fontan patients will become more clear. This will result in a more appropriate selection of patients that will profit most of the use of ACE inhibitors.

## 2. Objectives

The objectives of the present study are as follows:

-To assess autonomic parasympathetic and sympathetic function as well as vascular function in a population of patients with univentricular hearts palliated by the Fontan procedure and compare it with the results in healthy subjects.

-To assess the effects of central fluid depletion by means of a passive head-up-tilt test and central fluid loading by passive leg raising on cardiac output, aortic distensibility and hepatic venous blood flow patterns in a group of patients with a Fontan circulation and compare it with the results in healthy controls.

-To correlate the above mentioned results with the results of a symptom limited maximal exercise test.

-To treat Fontan patients for 3 months with the ACE inhibitor enalapril and compare a set of cardiovascular measurements before and after treatment in order to study its effect on the cardiovascular system in Fontan patients.

-To study the effect of a reversible fluid challenge and fluid depletion on cardiovascular parameters before and after a period of enalapril treatment in order to study changes in fluid susceptibility in Fontan patients.

**3. Study design**

In the present study we will compare several cardiovascular measurements (described below at '6. Study parameters') before and after treatment of enalapril, in patients with a univentricular heart after palliation with the Fontan circulation. Patients will start with treatment of enalapril after all cardiovascular measurements at baseline have been performed. After a 3-month period of treatment with enalapril, all cardiovascular measurements will be repeated.

Healthy age and gender matched subjects will serve as controls. All cardiovascular measurements, except for blood testing and cardiopulmonary exercise stress testing, will be performed just once in healthy controls. They will not be treated with enalapril.

**4. Study population**

Population:

Patients, who have been operated in the Leiden University Medical Centre (LUMC), with a univentricular heart after palliation with the Fontan circulation will be included. We strive for including 55 patients. A number of 55 healthy age and gender matched subjects will serve as controls.

Inclusion criteria:

Patients with a univentricular heart after palliation with the Fontan circulation from 8-18 years old.

Exclusion criteria:

Patients who already use enalapril.

Sample size calculation:

To detect statistical significant difference of our main study parameter $VO_2$peak (ml/kg/min), between Fontan patients before and after treatment with enalapril, we calculated a group size of 52 will be sufficient. We calculated this sample size for paired observations with a clinical relevant effect of 10% elevation after treatment, with a mean of 33.5 and SD of 6.8 before treatment (Bossers et al.; J Thorac Cardiovasc Surg 2014) and an elevation of the mean to 36.85 with an expected same SD after treatment and calculated a SD of the difference of 6.8 with a correlation coefficient of 0.5. We used an alpha of 0.01 with 80% power. We used a lower alpha of 0.01 to correct for multiple comparison according to the Bonferroni correction, because we have five main study parameters. Moreover, we also need to take the change of drop out into account, which means we need 55 patients per group when we expect a 5% drop out. This means a group size of in total 55 Fontan patients and a group of 55 healthy subjects will be sufficient.

**5. Treatment of subject:**

To all Fontan patients enalapril will be given twice daily at a dose of 0,5 mg/kg/day with a maximum of 20 mg per day. Enalapril will be titrated on average in two steps, by blood pressure measurements, which will be measured by the general practitioners of the patients. When a fall of more than 20% of the systolic blood pressure occurs, enalapril dosage will be lowered to a maximum acceptable dosage at which a fall of more than 20% of the systolic blood pressure does not occur. Dosage will also be lowered when Fontan patients feel light-headed or have complaints of dizziness. After a period of three months all procedures that are performed at the start of the study will be repeated.

This is a study of medicinal products within the meaning of the “WMO”. Which means preparation and labelling of the investigational medicinal products will be done according to the relevant GMP guidelines. The medicinal products in this study will be labelled and issued by the hospital pharmacy of the LUMC, which has a GMP license.

**6. Study parameters**

Main study parameters:

- *Cardiopulmonary exercise stress test*: $VO_2$peak.

- *Cardiac autonomic nervous activity*: heart rate variability and pre-ejection period.

- *Outcome of passive leg raising and head up tilt table testing*: cardiac output and cardiac autonomic tone.

Secondary study parameters:

- *Echocardiography*: Complete echocardiographic evaluation will be performed. This includes 2D imaging, colour Doppler and pulse wave velocity measurements of inflow and outflow through the cardiac valves. Tissue Doppler imaging as well as speckle tracking strain imaging will be performed. Special attention will be paid to hepatic venous blood flow patterns and superior caval venous (Glenn) flow patterns.

- *Cardiopulmonary Exercise Stress Testing*: A symptom limited maximal exercise stress test will be performed on a bicycle ergometer. During the stress test heart rate, and carbon dioxide production will be measured. This enables the assessment of maximal oxygen consumption, $VE/VCO_2$ relationship and the measurement of the Oxygen Uptake Efficiency Slope (OUES).

- *Blood Sampling*: From a venous puncture blood will be taken to assess electrolytes (Na and K), kidney function (Creatinine and Urea), liver function (ASAT, ALAT, alkalic phosphatase, gamma globulin and bilirubin levels), albumin and NT-pro BNP levels.

- *24-hour monitoring of electrocardiography and impedance cardiography*: To monitor ECG and ICG non-invasively, continuously during 24 hours we will use the VU-AMS device (De Geus et al 1995; Riese et al 2003; Van Lien et al 2013; Willemsen et al 1996). By the use of seven electrodes on the thorax this ambulatory device continuously records the electrocardiogram, impedance cardiogram and movement (by means of a three axial accelerometer). VU-AMS makes it possible to monitor non-invasively in a continuous way during daily life activities heart rate, heart rate variability, stroke volume and pre ejection period. Respiratory sinus arrhythmia (derived from the electrocardiogram and respiration) is a measure of the cardiac parasympathetic activity; the pre ejection period (derived from combining electrocardiogram and impedance cardiogram signals) is an index of cardiac contractility and a reflection of cardiac sympathetic control. Respiratory sinus arrhythmia measured by the VU-AMS is based on the peak-valley method. The time between two successive R peaks in the electrocardiogram (the inter beat interval) is calculated by the VU-AMS software. The difference between the shortest interval during inspiration and the longest interval during exhalation is defined as the respiratory sinus arrhythmia and is used as a measure of cardiac vagal

tone, representing the parasympathetic nervous activity. Pre-ejection period is defined as the time between the onset of the depolarization of the ventricles (reflected by the Q-onset in the electrocardiogram) and the opening of the aortic valves (reflected by the B-point in the impedance cardiogram)  In addition, the impedance cardiogram can be used to assess stroke volume (changes).-

- *Aortic stiffness*: By means of the arteriograph the pulse wave velocity of the aorta can be measured (Horvath et al 2010). This measurement is a surrogate of aortic stiffness. By means of the arteriograph, pulse wave velocity, augmentation index and central blood pressure can be measured non-invasively in a reliable and easy way.

- *Outcome of passive leg raising and head up tilt table testing:* After the baseline cardiovascular parameters have been performed the patient will undergo a passive leg raising test and a head up tilt table testing. There will be a period of at least 30 minutes between the two tests to allow the circulation to return to baseline levels.

- Passive leg raising: By means of passive leg raising an easy, safe and reversible fluid load can be given. After stabilization aortic pulse wave velocity will be measured by the arteriograph, while using echocardiography changes inand hepatic venous blood flow can be measured. Cerebral blood flow will be measured by Doppler recordings.
- Head up tilt table testing: Passive head-up-tilt testing induces an easily and fast reversible unloading of the central blood volume. After stabilization aortic distensibility will be measured by the arteriograph, while using echocardiography changes in hepatic venous blood flow can be measured. Cerebral blood flow will be measured by Doppler recordings. A Finapres device will be used to continuously non-invasively measure blood pressure.

**7. Data analysis**

The study has a cross-sectional between-subject design paired with prospective within-subject design. Baseline cardiovascular measurements will be compared between the Fontan patients and their age and gender matched healthy controls. Comparisons between the groups will be made using the independent T-test in case of normal distribution and Mann-Whitney U test in case of non-normal distribution (SPSS for Windows, recent version).

After a period of 3 months treatment with enalapril all baseline cardiovascular measurements will be repeated and compared to pre-treatment values (echocardiography, cardiopulmonary exercise stress testing, blood testing, 24-hour monitoring of electrocardiography and impedance cardiography, and aortic distensibility). Also the fluid challenge by passive leg raising as well as the depletion of central and splanchnic blood volume by head up tilt testing will be repeated and compared to pre-treatment values.

Statistics

In general data will be expressed as mean (standard deviation) in case of normal distribution, or median (interquartile range) in case of non-normal distribution.

**8. Expected results**

- In this study we expect to find a more prominent effect of fluid challenges on cardiovascular parameters in patient with a Fontan circulation as compared to healthy controls.
- We expect that the use of enalapril in Fontan patients will exaggerate the responsiveness to central blood volume depletion and will increase the responsiveness to fluid challenge as well.
- We expect that the use of enalapril will result in a decreased aortic pulse wave velocity, a better cardiac autonomic profile and an increase in cardiopulmonary exercise stress testing.
- By evaluating the basic characteristics of patients who respond best on enalapril treatment with those who respond the least will probably result in the identification of a basic profile of those patients who will profit best of enalapril treatment.

## 9. Administrative aspects of participants data

All the data of all participants that will be collected during this study will be treated anonymously and confidentially.

Confidentially of participants data

Each study subject will receive a specified code that will be connected to the study data. This will be done to protect the privacy of each participant. This specific code will not consist of personal information that can lead to the study participant. Only the responsible- and principle investigators will have the key of the code to know which code stands for which participant. The key of this code is safely stored at the local institution where the research is carried out. Data of this study can be used in reports or scientific publications, but data will then still no be traceable to individual participants.

Access to data of participants

Several persons are allowed to access the medical and personal records. This is needed to verify that this research project is carried out in a good and reliable way. The people allowed to access these records are: the research team, the monitors that monitor this research and the Dutch "Health and Youth Care Inspectorate. All these persons will keep the records of the participants secret. Non-authorised outsiders will not have access to these records.

Retention period of participants data

All data from this research will be collected and then stored for at least 15 years.

Information about unexpected results

During this research it is possible that accidently something is found that is not important for the research, but is important for the healthcare of the participant. In this case the concerning participant will be informed. The participant can discuss with his or her treating paediatric cardiologist or general practitioner what has to be done with these results. The participant gives permission for this.

Withdrawal of consent

The participant can always withdraw the consent for this research. When a participant withdraws the consent, already collected data will still be used for the research.

More information about rights and processing of participants data

For general information about participant's rights at processing participant's data, the website of the Dutch authority of personal data or Leiden University Medical Centre can be consulted. Participants can contact the principal investigator for questions about their rights. The principal investigator is Dr. A.D.J. ten Harkel, Tel: +3171-5262835, E-mail: A.D.J.ten_Harkel@lumc.nl. Participants who have questions or complaints about processing of personal data can contact the research location first. They can also contact the officer of data protection of the LUMC (Yvonne Zegers, E-mail: infoavg@lumc.nl, Albinusdreef 2, 2333 ZA Leiden; central phone number: +31715269111) or the Dutch authority of personal data.

Registration of this research

Information about this research is also included in an overview of medical-scientific research, called the Dutch trial registry (http://www.trialregister.nl/trialreg/index.asp). Data about this research on this website is not traceable to individual participants. After the research has been ended, this website can give a summary of the results. This research can be found under: "ACE inhibition in Fontan patients: its effect on body fluid regulation"; acronym: SAFE, NTR number: NTR6591.

**10. References**

Abraham MR, Olson LJ, Joyner MJ, Turner ST, Beek KC, Johnson BD. Angiotensin converting enzyme genotype modulates pulmonary function and exercise capacity in treated patients with congestive stable heart failure. Circulation 2002;106:1794-1799.

Amjad A. Kouatli, Jorge A. Garcia, Thomas M. Zellers, Ellen M. Weinstein and Lynn Mahony Enalapril Does Not Enhance Exercise Capacity in Patients After Fontan Procedure. Circulation 1997;96:1507-1512.

Beekman, R. H.; Katz, B. P.; Moorehead-Steffens, C.; Rocchini, A. P. Altered baroreceptor function in children with systolic hypertension after coarctation repair. *Am. J. Cardiol.* **1983,** *52* (1), 112-117

Biglino G, Schievano S, Steeden JA, Ntsinjana H, Baker C, Khambadkone S, et al. Reduced ascending aorta distensibility relates to adverse ventricular mechanics in patients with hypoplastic left heart syndrome: noninvasive study using wave intensity analysis. J Thorac Cardiovasc Surg 2012;144:1307-1314.

Bossers SS, Helbing WA, Duppen N, Kuipers IM, Schokking M, Hazekamp MG, Bogers AJ, Ten Harkel AD, Takken T. Exercise capacity in children after total cavopulmonary connection: lateral tunnel versus extracardiac conduit technique. J Thorac Cardiovasc Surg 2014;148:1490-1497.

Bossers SSM, Duppen N, Kapusta L, Maan AC, Duim AR, Bogers AJJC, et al. Comprehensive rhythm evaluation in a large contemporary Fontan population. J Cardiothorac Surg 2015;48:833-841.

Burchill LJ, Redington AN, Silversides CK, Ross HJ, Juan HJ, Mital S, et al. Renin-angiotensin-aldosterone system genotype and serum BNP in a contemporary cohort of adults late after Fontan palliation. Int J Cardiol 2015;197:209-215.

Chen RH, Wong SJ, Wong WH, Cheung YF. Arterial mechanics at rest and during exercise in adolescents and young adults after arterial switch operation for complete transposition of the great arteries. Am J Cardiol 2014;113:713-8.

Cherpanath TGV, Hirsch A, Geerts BF, Lagrand WK, Leeflang MM, Schultz MJ. Groeneveld ABJ. Predicting Fluid Responsiveness by Passive Leg Raising: A Systematic Review and Meta-Analysis of 23 Clinical Trials. Crit Care Med 2016;44:981-991.

Chrysohoou C, Skoumas J, Oikonomos E, Tsiachris D, Metaxa V, Lagoudakou S, et al. Aortic artery distensibility shows inverse correlations with heart rate variability in elderly non-hypertensive, cardiovascular disease-free individuals: the Ikaria study. Heart Vessels 2013;28:467-472

Contini M, Compagnino E, Cattadori G, Magri D, Camera M, Apostolo A, et al. ACE-inhibition benefit on lung function in heart failure is modulated by ACE insertion/deletion polymorphism. Cardiovasc Drugs Ther 2016;30:159-168.

Dahlqvist JA, Karlsson M, Wiklund U, Hornsten R, Stromvall-Larsson E, Berggren H, et al. Heart rate variability in children with Fontan circulation: lateral tunnel and extracardiac conduit. Pediatr Cardiol 2011;33:307-15.

De Devitiis M, Pilla C, Kattenhorn M, Zadinello M, Donald A, Leeson P, et al. Vascular dysfunction after repair of coarctation of the aorta ; impact of early surgery. Circulation 2001;104 Suppl I: I-165-I-170.

De Geus EJ, Willemsen GH, Klaver CH, van Doornen LJ. Ambulatory measurement of respiratory sinus arrhythmia and respiration rate. Biological Psychology 41: 205-27, 1995.

Dekker JM, Schouten EG, Klootwijk P, Pool J, Swenne CA, Kromhout D. Heart rate variability from short electrocardiographic recordings predicts mortality from all causes in middle-aged and elderly men. The Zutphen Study. Am J Epidemiol 145: 899-908, 1997.

De Mey W, Cools B, Heying R, Budts W, Louw JJ, Boshoff DE, Brown SC, Gewillig M. Can a volume challenge pinpoint the limiting factor in a Fontan circulation? Acta Cardiol. 2015;70:536-42.

Falkenberg C, Östman-Smith I, Gilljam T, Lambert G, Friberg P. Cardiac autonomic function in adolescents operated by arterial switch surgery. Int J Cardiol 2013;168:1887-93.

Gewillig M, Brown SC. The Fontan circulation after 45 years, update in physiology. Heart 2016.

Groehs RV, Toschi-Dias E, Antunes-Correa LM, Trevizan PF, Urbana M, Rondon PB, et al. Exercise training prevents the deterioration in the arterial baroreflex control of sympathetic nerve activity in chronic heart failure patients. Am J Physiol Heart Circ Physiol 2015;308:H1096-H1102.

Haggerty CM, Whitehead KK, Bethel J, Fogel MA, Yoganathan AP. Relationship of single ventricle filling and preload to total cavopulmonary connection hemodynamics. Ann Thorac Surg 2015;99:911-917.

Hidvegi EV, Illyes M, Benczur B, Bocskei RM, Ratgeber L, Lenkey Z et al. Reference values of aortic pulse wave velocity in a large healthy population aged between 3 and 18 years. J Hypertens 2012;30:2314-21.

Horvath IG, Nemeth A, Lenkey Z, Alessandn N, Tufano F, Kis P, Gaszner B, Cziraki A. Invasive validation of a new oscillometric device (arteriography) for measuring augmentation index, central blood pressure and aortic pulse wave velocity. J Hypert 2010;28:2066-2078.

Hsia TY, Khambadkone S, Deanfield JE, Taylor JFN, Migliavacca F, De Leval MR. Subdiaphragmatic venous hemodynamics in the Fontan circulation. J Thorac Cardiovasc Surg 2001;121:436-447.

Hsu DT, Zak V, Mahony L, Sleeper LA, Atz AM, Levine JC, et al. Enalapril in infants with single ventricle; results of a multicentre randomized trial. Circulation 2010;122:333-340.

Kenny D, Polson JW, Martin RP, Caputo M, Wilson DG, Cockcroft JR, Paton JF, Wolf AR. Relationship of aortic pulse wave velocity and baroreceptor reflex sensitivity to blood pressure control in patients with repaired coarctation of the aorta. Am Heart J 2011;162:398-404.

Kenny, D.; Polson, J. W.; Martin, R. P.; Paton, J. F.; Wolf, A. R. Normalization of autonomic function in children with coarctation of the aorta after surgical correction in infancy. *Hypertension* **2009,** *54* (3), e21-e22

Kojima T, Kuwata S, Kurishima C, Iwamoto Y, Saiki H, Ishido H, et al. Aortic root dilatation and aortic stiffness in patients with single ventricular circulation. Circ J 2014;78:2507-2511.

La Gerche A, Gewillig M. What Limits Cardiac Performance during Exercise in Normal Subjects and in Healthy Fontan Patients? Int J Pediatr 2010;

Lam Y-Y, Mullen MJ, Kaya MG, Gatzoulis MA, Li W, Henein MY. Left ventricular long axis dysfunction in adults with “corrected” aortic coarctation is related to an older age at intervention and increased aortic stiffness. Heart 2009;95:733-739.

Lambert E, d’Udekem Y, Cheung M, Sari CI, Inman J, Ahimastos A, et al. Sympathetic and vascular dysfunction in adult patients with Fontan circulation. Int J Cardiol 2013;167:1333-1338.

La Rovere MT, Pinna GD, Hohnloser SH, Marcus FI, Mortara A, Nohara R, Bigger JT, Camm AJ,

Schwartz PJ. Baroreflex sensitivity and heart rate variability in the identification of patients at risk for life-threatening arrhythmias - Implications for clinical trials. Circulation 103: 2072-7, 2001.

Lee KJ, Yoo SJ, Holtby H, Grant B, Mroczek D, Wong D, et al. Acute effects of the ACE inhibitor enalapril on the pulmonary, cerebral and systemic blood flow and resistance after the bidirectional cavopulmonary connection. Heart 2011;97:1343-1348.

Maida KD, De Souza D. Effects of treatment with enalapril or losartan associated with aerobic physical training on cardiovascular autonomic control in spontaneously hypertensive rats (SHR). J Hypertension2015;33 Suppl 1:e78.

Millar PJ, Proudfoot NA, Dillenburg RF, Macdonald MJ. Reduced heart rate variability and baroreflex sensitivity in normotensive children with repaired coarctation of the aorta. Int J Cardiol 2013;168:587-8.

Moutafi AC, Manis G, Dellos C, Tousoulis D, Davos CH. Cardiac autonomic nervous activity in adults with coarctation of the aorta late after repair. Int J Cardiol 2014;173:566-8

Myers KA, Leung MT, Potts T, Potts JE, Sandor GGS. Noninvasive assessment of vascular function and hydraulic power and efficiency in pediatric Fontan patients. J Am Soc Echocardiogr 2013;26:1221-7.

Nemes A, Takacs R, Gavaller H, Varkonyl TT, Wittmann T, Forster T, Lengyei C. Correlations between aortic stiffness and parasympathetic autonomic function in healthy volunteers. Can J Physiol Pharmacol 2010;88:1166-1171.

Nieminen HP, Jokinen EV, Sairanen HI. Causes of late deaths after pediatric cardiac surgery: a population-based study. J Am Coll Cardiol 2007 Sep 25;50(13):1263-71.

Nolan J, Batin PD, Andrews R, Lindsay SJ, Brooksby P, Mullen M, Baig W, Flapan AD, Cowley A, Prescott R, Neilson J, Fox K. Prospective study of heart rate variability and mortality in chronic heart failure: results of the United Kingdom heart failure evaluation and assessment of risk trial (UK-heart). Circ J 1998;98:1510-1516.

Ohuchi H, Ono S, Tanabe Y, Fujimoto K, Yagi H, Sakaguchi H, Miyazaki A, Yamada O. Long-term serial aerobic exercise capacity and hemodynamic properties in clinically and hemodynamically good, excellent, Fontan surviviors. Circ J 2012;76:195-203.

Ohuchi H· Adult patients with Fontan circulation: What we know and how to manage adults with Fontan circulation? J Cardiol 2016;68:181-9.

Ohuchi H, Takasugi H, Ohashi H, Okada Y, Yamada O, Ono Y, Yagihara T, Echigo S. Stratification of pediatric heart failure on the basis of neurohormonal and cardiac autonomic nervous activities in patients with congenital heart disease. Circulation. 2003 Nov 11;108(19):2368-76.

Ohuchi H, Negishi J, Miyake A, Sakaguchi H, Miyazaki A, Yamada O. Long-term prognostic value of cardiac autonomic nervous activity in postoperative patients with congenital heart disease. Int J Cardiol. 2011 Sep 15;151(3):296-302.

Ono M, Kasnar-Samprec J, Hager A, Cleuziou J, Burri M, Langenbach C, Callegari A, Strbad M, Vogt M, Hörer J, Schreiber C, Lange R. Clinical outcome following total cavopulmonary connection: a 20-year single-centre experience. Eur J Cardiothorac Surg. 2016

Ou P, Celermajer DS, Jolivet O, Buyens F, Herment A, Sidi D, et al. Increased aortic stiffness and left ventricular mass in normotensive young subjects after successful coarctation repair. Am Heart J 2008;155:187-193.

Polson, J. W.; McCallion, N.; Waki, H.; Thorne, G.; Tooley, M. A.; Paton, J. F.; Wolf, A. R. Evidence for cardiovascular autonomic dysfunction in neonates with coarctation of the aorta. *Circ* **2006,** *113* (24), 2844-2850

Riese H, Groot PFC, van den Berg M, Kupper NHM, Magnee EHB, Rohaan EJ, Vrijkotte TGM, Willemsen G, de Geus EJC. Large-scale ensemble averaging of ambulatory impedance cardiograms. Behavior Research Methods Instruments & Computers 35: 467-77, 2003.

Robbers-Visser D, Ten Harkel ADJ, Kapusta L, Strengers JL, Dalinghaus M, Meijboom FJ, Pattynama PM, Bogers AJ, Helbing WA. Usefulness of cardiac magnetic resonance imaging combined with low-dose dobutamine stress to detect an abnormal ventricular stress response in children and young adults after fontan operation at young age. AM J Cardiol. 2008 Jun 1;101(11):1657-62.

Saul JP, Arai Y, Berger RD, Lilly LS, Colucci WS, Cohen RJ. Assessment of autonomic regulation in chronic congestive heart failure by heart rate spectral analysis. American Journal of Cardiology 61: 1292-9, 1988.

Schwartz PJ, La Rovere MT, De Ferrari GM, Mann DL. Autonomic modulation for the management of patients with chronic heart failure. Circ Heart Fail 2015;8:619-628.

Schwartz PJ, La Rovere MT, Vanoli E. Autonomic nervous system and sudden cardiac death. Experimental basis and clinical observations for post-myocardial infarction risk stratification. Circulation 85: I77-I91, 1992.

Szczepaniak-Chichel L, Trojnarska O, Mizia-Stec K, Gabriel M, Grajek S, Gasior Z, et al. Augmentation of central arterial pressure in adult patients after coarctation repair. Blood Pressure Monitoring 2011;16:22-28.

Tomkiewicz-Pajak L, Dziedzic-Oleksy H, Pajak J, Olszowska M, Kolcz J, Komar M, Podolec P. Arterial stiffness in adult patients after Fontan procedure.  Cardiovasc Ultrasound 2014;12:15.

Vanderschuren MM, Uiterwaal CSPM, Van der Ent CK, Eising JB. Feasibility and characteristics of arterialstiffness measurement in preschool children (Submitted)

Van Lien R, Schutte NM, Meijer JH, de Geus EJ. Estimated preejection period (PEP) based on the detection of the R-wave and dZ/dt-min peaks does not adequately reflect the actual PEP across a wide range of laboratory and ambulatory conditions. International Journal of Psychophysiology 87: 60-9, 2013.

Voges I, Jerosch-Herold M, Hedderich J, Hart C, Petko C, Scheewe J, Andrade AC, Pham M, Gabbert D, Kramer HH, Rickers C. Implications of early aortic stiffening in patients with transposition of the great arteries after arterial switch operation. Circ Cardiovasc Imaging 2013;6:245-53.

Willemsen GH, de Geus EJ, Klaver CH, van Doornen LJ, Carroll D. Ambulatory monitoring of the impedance cardiogram. Psychophysiology 33: 184-93, 1996.

Wilson TG, Iyengar AJ, Winlaw DS, Weintraub RG, Wheaton GR, Gentles TL, et al. Use of ACE inhibitors in Fontan: rational or irrational? Int J Cardiol 2016;210:95-99.

Wilson TG, Iyengar AJ, d'Udeken Y. The use and misuse of ACE inhibitors in patients with single ventricle physiology. Heart Lung Circ 2016;25:229-236.

Yim DLS, Jones BO, Alexander PMA, d'Udeken Y, Cheung MMH. Effect of anti-heart failure therapy on diastolic function in children with single-ventricle circulations. Cardiol Young 2015;25:1293-1299.
